# The Road to Maternal Death In Rural Southwest Ethiopia

**DOI:** 10.4314/ejhs.v20i1.69423

**Published:** 2010-03

**Authors:** Kebede Deribe, Sibhatu Biadgilign, Alemayehu Amberbir, Tefera Belachew, Kifle Woldemichael

**Affiliations:** 1 FIDO Ethiopia, P.O. Box 1876/1250, Addis Ababa, Ethiopia, *e-mail:*kebededeka@yahoo.com; 2 John Hopkins University-Technical Support for the Ethiopian HIV/AIDS ART Initiative, Addis Ababa, Ethiopia; 3 Addis Ababa University, School of public health, Butajera Birth Cohort study; 4 Jimma University, Population and Family Health Department, Jimma University, P.O. Box: 1104, Jimma, Ethiopia. E-mail: tefera_belachew@yahoo.com; 5 Department of Biostatistics and Epidemiology, Jimma University

## Abstract

The study explored cultural beliefs and practices contributing to maternal deaths together with maternal deaths reviews as testimonial. Six maternal deaths were retrospectively observed in rural southwest Ethiopia. Four of the 6 deaths occurred due to direct obstetric causes. Substandard primary and referral care, not understanding the severity of the problem, and lack of transport were the major themes identified as contributing factors. The result highlighted the need to improving primary health care, to strengthen referral system and community education.

## Introduction

It was estimated that maternal deaths in 2000 for the world was 529,000. Thirteen countries including Ethiopia account for 67% of all maternal deaths (1). According to the 2000 estimate, Ethiopia ranks 4^th^ (24,000) in the absolute number and 22^nd^ in maternal mortality ratio (MMR) (1). The current estimate for MMR in Ethiopia is 673 per100, 000 live births (2). This is one of the highest in the world and demands in-depth understanding of reasons for high maternal mortality.

Researches done so far tried to quantify the number and causes of maternal deaths (3–5). Knowing the level of maternal mortality is not enough; we need to understand the underlying factors that led to the deaths. Each maternal death has a story to tell and can provide indications on practical ways of addressing its causes and contributing factors (6). It also provides a unique opportunity to include the family and community's opinion on the access to and the quality of health services (7, 8). Therefore, this study explored cultural beliefs and practices contributing to maternal deaths.

## Methods

A qualitative study was conducted from November 2 to 30, 2006 at Gilgel Gibe filed research center. The Gilgel Gibe Hydroelectric dam is located 260 K.M Southwest of Addis Ababa and 70 K.M Northeast of Jimma.

According to baseline survey done in August 2005, there were a total of 8859 households and 42,290 individuals in the area. Most, 94.4% of the deliveries happen at home commonly attended by neighbors (67.5%), relatives (20.5%) and traditional birth attendants (4.7%).

Sixty seven percent of women in the reproductive age had heard about modern family planning methods. Among these, 19.8% have ever used modern family planning methods and 18% are currently using modern family planning methods (9).

Exploratory qualitative method was used to identify factors related to maternal death. Six maternal deaths were identified using snowball method. Data were collected about the cause, individual behaviors and things surrounding maternal death from the husband of the deceased. In the events where the husband is not found, grandmothers and other relatives/neighbors were asked.

Four focus group discussions (FGDs) involving 6–12 people each were held with women, grandmothers, and traditional birth attendants to identify cultural practices and beliefs, which could contribute to maternal deaths.

## Results

### Pregnancy and its interpretation

Pregnancy is considered in the area as a special event which brings a special respect and prestige to a woman. However, it was also experienced as time at which women live with uncertainty. In the area, delivery related complications are feared and women pass all those nine months between a feeling of life and death. Pregnant women were exempted from their routine work. There was no any additional food which is given to the pregnant women beyond the routine family food. There is a practice of confining women for 40 days post partum, which is the time when she will have a special care and feeding.

Abdominal massaging and skin piercing were also practices during delivery to reposition the fetus and to relief abdominal pain. Placenta is considered to be retained if it is not delivered within one hour after delivery.

Only few of the respondents and discussants clearly described the major danger sign of pregnancy. While probing, the most danger signs reported by these women were mal-presentation, profuse bleeding, delay in delivery and retained placenta. Other danger signs are not recognized by pregnant women and TBAs. Slight bleeding during pregnancy is considered as normal. If women complain such kinds of bleeding the elderly will tell that this is normal. Swelling of leg and face were considered to be minor problems which could be relived by delivery.

### Maternal death

Six deaths were reported between September 2003 and September 2006. For four of the deaths, we were able to interview one or more individuals present during their death. Four of six deaths occurred due to direct obstetric causes of death, which were hemorrhage (2); hypertensive disease of pregnancy (1) and abortion (1). There were 2 deaths by indirect obstetric causes, which were due to malaria which is endemic to the area and gestational diabetes. Two women died during pregnancy, one during labor and three during postpartum period. All except one died at home.

### Factors Contributing to maternal death

Substandard primary care in giving appropriate Ante Natal Care, Substandard referral care in giving adequate information for the people to understand the severity of the problem, and lack of transport were the major factors identified as contributing. In addition, failure to recognize the severity of the problem leading to delay in starting decision making to seek health care and lack of access to obstetric care were also additional factors contributing to maternal deaths.

## Discussion

In this study substandard referral care was found to be the major contributing factor to maternal death. The health workers were not telling the severity of the situation or they tend to ignore danger signs which could require immediate referral. Similarly such types of report were found in other studies [10]. The role of appropriate antenatal care is to address issues related to maternal and child health services. Improved technical skills in identifying danger signs as well as skills in information, communication, and early advice on referral are important [13].

The other most important contributing factor identified in this study was that families of the deceased did not understand the severity of the situation which leads to delay in seeking care. Studies had identified that recognition of an illness may be influenced by factors as the occurrence of the condition [14]. In one African study women considered fever, pallor and dizziness as normal signs of pregnancy because these conditions were common in that area [15]. In addition, studies have shown that there is a much higher threshold of tolerance to pain among individuals who live in extreme poverty than among those of the middle class[11]. This could be the reasons why many women or their relatives opted to do nothing in spite of the emergence of danger signs during their pregnancy. Transportation difficulties were also mentioned as contributing factors similar to other studies [6, 10, 12]. In most instances the community in the area lacks regular public transportation. Cultural practices to deliver placenta and confinement after delivery would also interfere with decisions to seek care. In conclusion substandard primary care, poor referral care, failure to recognize the severity of the problem leading to delay in starting decision making to seek health care and lack of access to obstetric care were factors contributing to maternal deaths in the area.

The study highlighted the need for improving the skills of primary health care workers, to strengthen referral system and availing ambulance service for the most at need community. In addition community based education tailored to local cultural practice focusing on danger signs and on the need to take prompt action in seeking care should be considered to reduce maternal death.

## Figures and Tables

**Table 1:** Main features of the maternal deaths in Gilge Gibe field research center, Southwest Ethiopia, 2006.

Age	Parity	ANC	Time of death	Birth outcome
27	2	Yes (4^th^)	8 months pregnancy	NA
27	6	No	20 days postpartum	live birth, alive
25	1	Yes	9 hours postpartum	Live birth, died at 2 weeks
28	2	No	Unknown duration of amenorrhea	NA
35	0	Yes	1 hour postpartum	live birth, died immediately
35	9	No	Died during labor	Died, undelivered

NA = Not applicable

**Table 2:** Causes and Contributing factors to maternal deaths in Gilgel Gibe field research center, Southwest Ethiopia, 2006.

Probable cause of death	Contributing factors	
	Probable	Possible
Indirect Causes of Death		
Malaria	Substandard primary care	Substandard referral care
Gestational diabetes	Substandard primary care Substandard referral care and information	Lack of transport Not recognized the severity of the problem
Direct Causes of Death		
Pre-eclampsia	Substandard primary care Substandard referral care information	Substandard referral care
Abortion(sepsis)	Substandard care	Substandard care
Hemorrhage, undetermined	Not recognized the severity of the problem Delay in starting decision making	Not recognized the severity of the problem
Hemorrhage, Undetermined	Not recognized the severity of the problem Delay in starting decision making	Delay in starting decision making
